# Voluntary movements as a possible non-reflexive pain assay

**DOI:** 10.1186/1744-8069-9-25

**Published:** 2013-05-20

**Authors:** Hawon Cho, Yongwoo Jang, Byeongjun Lee, Hyeyoun Chun, Jooyoung Jung, Sung Min Kim, Sun Wook Hwang, Uhtaek Oh

**Affiliations:** 1 Sensory Research Center, CRI, College of Pharmacy, Seoul National University, Seoul 151-742, Korea; 2 College of Physical Education, Hanyang University, Seoul 133-791, Korea; 3 Korea University Graduate School of Medicine, Seoul 136-705, Korea

**Keywords:** Voluntary movement, Rearing, Total distance moved, Inflammatory pain, Neuropathic pain, Visceral pain, TRPV1

## Abstract

**Background:**

The quantification of pain intensity *in vivo* is essential for identifying the mechanisms of various types of pain or for evaluating the effects of different analgesics. A variety of behavioral tests for pain measurement have been devised, but many are limited because animals are physically restricted, which affects pain sensation. In this study, pain assessment was attempted with minimal physical restriction, and voluntary movements of unrestrained animals were used to evaluate the intensities of various types of pain.

**Results:**

The number of times animals reared or total distances traveled was measured using a motion-tracking device and found to be markedly reduced in carrageenan-induced inflammatory, acetic acid-induced visceral, and streptozotocin-induced neuropathic pain tests. These two voluntary movement parameters were found to be highly correlated with paw withdrawal latency from irradiating heat. In addition, these parameters were markedly reversed by morphine and by non-steroidal anti-inflammatory drugs in inflammatory pain models. These parameters were also useful to detect hypoalgesia in TRPV1^-/-^ mice.

**Conclusions:**

These results suggest that parameters of voluntary movement, such as, number of rearing and total distance moved, are effective indicators of pain intensity for many types of pain and that they can be used to evaluate degree of pain perception.

## Background

Pain is an unpleasant sensation that warns of tissue damage, whether caused physically or chemically, and thus, the ability to detect pain is essential for protection and survival [1-3]. Whereas acute pain is evoked by damaging or intense stimuli, chronic pain is associated with injury and diseases [4,5]. Because chronic diseases, such as cancer, often accompany with severe pain, study of the pain mechanisms is essential to develop cures for chronic pain [6]. Because pain is a subjective, emotional, and pseudo-affective sensation, the estimation of pain intensity is substantially affected by emotional state. Many different types of pain measuring methods have been devised to measure pain-related behaviors elicited in animals to various insults [7-9]. These tests are largely based on the reflex responses of animals evoked by noxious or non-noxious stimuli. Common reflexive tests used to assess inflammatory or neuropathic pain are hot-plate, tail-flick, Hargreaves, Von Frey hair, and Randal Selitto tests [8-11]. These tests are useful tools in pain research and have been used widely. However, they may have some limitations. Specifically, in order to evoke reflex responses, various types of noxious or non-noxious stimuli need to be imposed on legs, tails or other parts of the body. Furthermore, free movement of an animal is somewhat restricted or placed in a small cage to deliver noxious stimuli. The need for physical restriction, by holding the trunk or legs or confining an animal to a small case, evidently creates stress, which is likely to affect pain sensitivity. In addition, reflex-based pain tests are also limited by their subjective nature of measurements. Measurement of withdrawals of tails or legs from noxious stimuli is sometimes ambiguous. Thus, it appears necessary to devise a pain test that circumvents these limitations.

In this study, we examined whether parameters based on voluntary movements could be used to assess pain severity objectively in specific pain models. We reasoned that unconfined mice or rats move freely and sometimes stand on their hind legs to explore their environments, but that animals in pain would be reluctant to move.

## Methods

### Animals

This study was performed in accordance with protocols approved by the Committee on Laboratory Animals at the Seoul National University. Experiments were also conducted according to the Ethical Guidelines of the International Association for the Study of Pain. Adult male Sprague–Dawley rats (180–230 g), wild type C57Bl/6J mice (18 – 23 g) (Koatech, Seoul), and TRPV1 deficient C57Bl/6J mice (Jackson Laboratory, Bar Harbor, ME) were used for the behavioral tests. All experiments were performed during the day time (9:00 a.m. and 6:00 p.m.). Animals for all experiments were habituated before testing.

### Carrageenan- induced inflammation

To induce inflammation, carrageenan (2%) was injected subcutaneously into both plantar surfaces of the hind paws of rats and mice (40 μl in rats and 20 μl in mice). For control experiment, 40 or 20 μl of saline were injected subcutaneously into both plantar surfaces of the hind paws of rats or mice.

### Drug treatment

Morphine (2 mg/kg or 10 mg/kg), ibuprofen (20 mg/kg) or diclofenac (10 mg/kg) were administered intraperitoneally (i.p.) 20 or 30 min or 1 hr before voluntary behavioral testing, respectively. Acetic acid (10 mg/kg; i.p.) or celecoxib (10 mg/kg; p.o.) were also administered to mice 15 min or 1 hr before voluntary behavioral test. All nonsteroidal anti-inflammatory drugs were purchased from Sigma-Aldrich and dissolved in physiological saline, with the exception of celecoxib that was suspended in 1% methylcellulose plus 0.1% tween80.

#### Spared nerve injury (SNI) model

Spared nerve injury was performed as previously described [12]. Briefly, mice were anesthetized by intraperitoneal injection of 50 mg/kg pentobarbital. We checked reflexes by pinching the paws using a pincette. Then, we used scissors to make a 1 cm incision in the longitudinal direction around the knee. Injury was induced in mice through cutting tibial and common peroneal nerve branches among three branches of the sciatic nerve. Same surgical procedure was performed in sham control, except that the sciatic nerve remained undisturbed.

### Streptozotocin-induced diabetes

Diabetes was induced in mice by administering streptozotocin (STZ) (150 mg/kg; i.p.). Plasma glucose levels were measured using a glucose test kit (LIFESCAN, Milpitas, CA) in blood samples obtained from a tail vein.

### Measurement of voluntary movements

An unrestrained mouse or rat was placed in a 43 cm × 43 cm activity cage and the number of rearings and the total distance traveled were measured using a fully automated, photocell-based system (ATM3 Auto-Track system, Columbus Instruments, Columbus, OH). This system involves multiple Opto-Varimex Animal activity monitors that are connected to a computer. The location of an animal in the horizontal plane (used to calculate the distance travelled) and number of beam breaks in the vertical plane (indicative of rearing), as determined by the Opto-Varimex optical tracking system, were transmitted to a computer using the Columbus Instruments Bus system. Horizontal activity was detected using 15 photo sensors on the front and back walls and 15 photo sensors on both side walls of the activity cage. Numbers of rearings were determined using 15 side wall sensors located 13 cm above the cage floor for rats and 6 cm for mice.

### Hargreaves plantar test

This test was performed using a standard apparatus (Ugo Basile Biological research Apparatus) as previously described [8]. Briefly, a rat was placed in a transparent acrylic box and infrared heat lamp was positioned underneath the targeted hind paw. A radiant stimulus was then applied to the plantar surface and paw withdrawal latency was measured.

### von Frey test

Mechanical allodynia was measured by prodding the plantar region of hindpaw with calibrated von Frey filaments (Stoelting Co., Wood Dale, IL). Animals were placed in cages with a mesh grid floor. On testing day, mice were allowed to acclimate for a minimum of 30 min before experiment. Plantar surface of hind foot was poked with von Frey filaments of different thickness. Withdrawal thresholds to von Frey filaments were determined when animals lifted a hindpaw at least 5 responses out of 10 stimulations. Minimum weight of the von Frey filament that evoked response was considered as a mechanical withdrawal threshold.

### Rotarod test

Animals were pre-trained on an automated 4-lane rotarod unit (Ugo basile, Italy) for 3 days before being treated with test drugs. The rotating rod was grooved to improve grip. An accelerating protocol was used. Briefly, a rat was placed on the rod, which was accelerated smoothly from 0 to 35 rpm over 3 minutes. Times on the rod were measured automatically by placing a trip switch under the floor beneath the rotating drum.

### Writhing test

Mice were injected with 10 ml/kg of 0.7% acetic acid i.p. and placed in an activity cage for observation. Numbers of writhes (defined as a contraction of the abdomen followed by twisting and turning of the trunk, and extension of hind limbs) were counted for 20 min.

### Statistics

All results are expressed as means ± SEMs, and were analyzed by one-way analysis of variance (ANOVA) followed by Tukey’s post-hoc test. Statistical significance was accepted for p values of < 0.05.

## Results

### Parameters of voluntary movement decrease during inflammation

**Figure 1:**
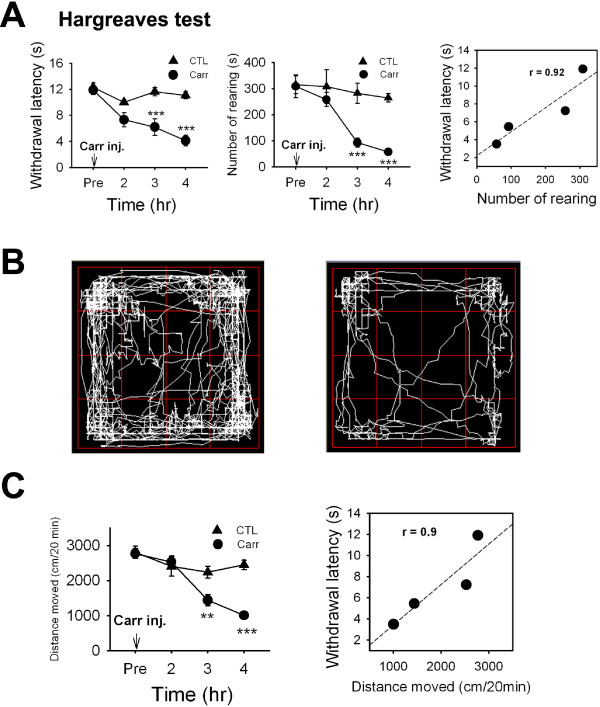
**Measurement of voluntary movement in a carrageenan-induced inflammatory pain model.** (**A**) *Left,* Carrageenan (2% in 40 μl) or saline was injected subcutaneously (s.c.) into the plantar surfaces of both hindpaws in rats. Latency of withdrawal response to a thermal stimulus was measured (n = 5–10, ***p<0.001) at 2h, 3h, 4h after carrageenan injection. Results are means ± SEMs. *Middle,* Number of rearings were measured at 2h, 3h, and 4h after carrageenan or saline injection for 20 min (n = 7–9). *Right,* Pearson correlation analysis of the relation between number of rearings and withdrawal latencies to noxious thermal stimuli in rats at 2h, 3h, and 4h after carrageenan injection (n = 5–9, *p<0.05, r^2^ = 0.92). (**B**) A trace of rat movements over 20 min after administering saline (CTL) (*left*) or carrageenan (Carr). (**C**) Total distances moved over 20 min were measured at 2h, 3h, and 4h after carrageenan or saline injection (n = 7–9). The correlation between total distance moved and withdrawal latency to noxious thermal stimuli was performed in rats 2h, 3h, and 4h after carrageenan injection (n = 5–9, *r*^*2*^ = 0.9).

To evaluate voluntary movements in relation to pain, rats were placed in the activity chamber and total distances travelled and number of rearings, which were considered a behavior related to curiosity or exploration [13,14], were recorded automatically (Figure 1). When saline-treated rats were placed in the activity chamber, they moved in a normal manner. Numbers of rearings and total distances moved in the activity cage over 20 min were counted and compared with the parameters measured using conventional reflexive pain tests.

First, Hargreaves test results and rearing behavior were compared using an inflammatory pain model. To induce inflammation, carrageenan (2% w/v, 40 μl; s.c.) was injected into the plantar surfaces of both hind paws of rats. For control experiment, physiological saline was administered into the plantar surfaces of both hind paws of rats. Withdrawal latency from radiant heat was measured 2, 3, and 4 hours after carrageenan or saline administration. As time passed, control group failed to show change in withdrawal latency. However, the withdrawal latencies of carrageenan-treated rats became significantly shorter than the latency before carrageenan administration (Figure 1A) with paw swelling and redness (data not shown). To obtain voluntary movement results, rats were placed in the activity chamber, and vertical and horizontal motions were recorded for 20 min at 2, 3, and 4 hours after carrageenan administration. As shown in Figure 1A, a marked time-dependent reduction in rearing behavior was observed, and this was found to be highly correlated with reductions in paw-withdrawal latency (Figure 1A, *r*^*2*^ = 0.92, n = 5–9).

The total distances moved by saline- and carrageenan administered rats were also evaluated. Figure 1B shows representative traces of saline- and carrageenan-treated rats free to explore the activity cage. The total distances moved over 20 min by carrageenan-treated rats 3 and 4 hours after carrageenan-injection were significantly lower than those of saline-treated rats (Figure 1C, n = 7–9). Total distances moved were also found to be highly correlated with paw withdrawal latencies determined by the Hargreaves test by linear regression (Figure 1C, *r*^*2*^ = 0.9, n = 5–9).

### Effects of nonsteroidal anti-inflammatory drugs on voluntary movements

**Figure 2:**
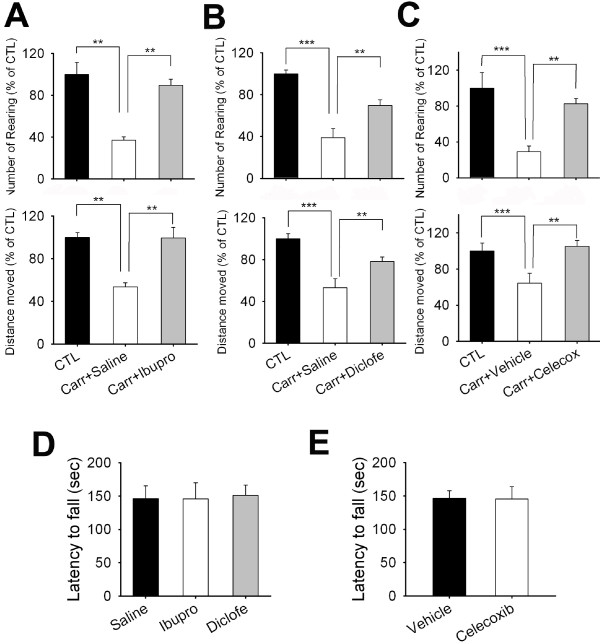
**The effects of nonsteroidal anti-inflammatory drugs on voluntary movements in carrageenan-injected rats.** (**A**) Numbers of rearings and the total distances moved over 20 min were measured at 4 h after carrageenan injection. Data represent the percentage of the numbers of rearing or total distances relative to their control values. Ibuprofen (20 mg/kg) and vehicle were administrated i.p. into rats 30 min before test (n = 7–8). (**B**) Numbers of rearings and the total distances moved over 20 min were measured at 4 h after carrageenan injection. Diclofenac (10 mg/kg) and saline were administrated i.p. into rats 1hr before test (n = 7–8). (**C**) Numbers of rearings and the total distances moved over 20 min were measured at 4 h after carrageenan injection. Celecoxib (10 mg/kg) and saline were administrated p.o. into rats before test (n = 7–8). **(D-E)** The effects of ibuprofen and diclofenac (**D**) and celecoxib (**E**) on motor function. Rotarod test was examined in rats treated with of ibuprofen (20 mg/kg), diclofenac (10 mg/kg), celecoxib (10 mg/kg), or vehicle. Time on the beam was recorded as latency (n = 7–9).

We then determined whether nonsteroidal anti-inflammatory drugs (NSAIDs) used commonly to treat inflammatory pain could recover the reduction of voluntary movements during inflammation. Ibuprofen (20 mg/kg) and diclofenac (10 mg/kg), non-selective cyclooxygenase inhibitors were injected intraperitoneally to carrageenan administered rats 30 min and 1 hr before measuring voluntary movements (4 hours after carrageenan administration). As shown in Figure 2A, ibuprofen-treated and carrageenan-injected rats moved as freely as treatment naïve rats. In contrast, saline treated and carrageenan-injected rats failed to move as vigorously as treatment naïve controls. Thus, reductions in total distance moved and numbers of rearings by carrageenan-induced inflammation were completely reversed by ibuprofen (Figure 2A). Diclofenac-treated and carrageenan-injected rats also recovered decrease of numbers of rearings and total distance moved induced by inflammation (Figure 2B). Next, we tested that the effect of a selective cyclooxygenase type 2 inhibitor, celecoxib on carrageenan injected-mice. As shown in Figure 2C, celecoxib (10 mg/kg, p.o.) and carrageenan treated mice significantly recovered the decrease of voluntary movements compared to vehicle and carrageenan treated mice. The reduction in voluntary movements was not due to the reduction in motor coordination because the NSAIDs-treated rats failed to exhibit a difference in time staying on rotating rod compared to saline or vehicle injected rats (Figure 2D, E).

### Effects of morphine on voluntary movements

**Figure 3:**
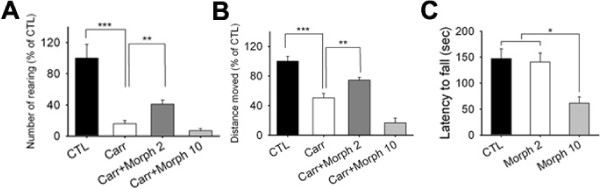
**The effects of morphine on voluntary movements in carrageenan-injected rats.** (**A**) Numbers of rearings were measured at 4h after carrageenan injection. Morphine was administered i.p. at 2 or 10 mg/kg 10 min before testing (n = 7–8). (**B**) Total distances moved over 20 min were measured 4h after carrageenan injection and 10 min after administering morphine (2 or 10 mg/kg, i.p.) (n = 7–8). (**C**) The effect of morphine on motor coordination was examined using the rotarod test in rats treated with 2 or 10 mg/kg of morphine. Times on the beam were recorded as latencies (n = 5–7).

Morphine administered to rats at 2 mg/kg (i.v.) significantly increased numbers of rearings and total distance moved versus carrageenan-injected rats (Figure 3A, B). However, when a maximum dose of morphine (10 mg/kg) was administered, the reduction in numbers of rearings and total distances was greater than after administering morphine at 2 mg/kg, suggesting a possible sedative effect. In fact, it has been well documented that a morphine dose greater than 5 mg/kg causes sedation [14,15]. Indeed, when rats administered 10 mg/kg morphine stayed on the rotating rod for less time than rats administered 2 mg/kg (140.9 ± 17.3 *vs* 61.6 ± 11.9 sec, p < 0.05, n = 5–7, Figure 3C), indicating a reduction in motor coordination or balance. This effect of sedation was not detected by conventional reflexive pain tests.

### Voluntary movements in mice with acute visceral pain

**Figure 4:**
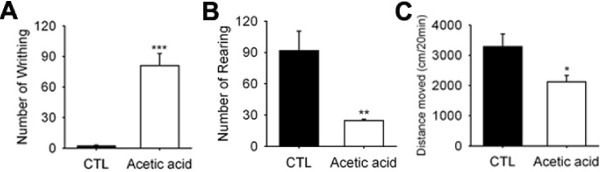
**Voluntary movements of mice with acute visceral pain induced by acetic acid.** (**A**) Acetic acid was administered at 10 mg/kg intraperitoneally. Numbers of writhes were measured over 20 min after the injection of saline or acetic acid (n = 6). (**B**-**C**) Voluntary movements were measured in saline or acetic acid treated mice. Acetic acid treated mice showed a significant reduction in number of rearings. (**B**) and total distance moved (**C**) (n = 6).

We now tested whether voluntary movements would also represent the acute pain state. To do so, we used an acute visceral pain model [16-18]. The administration of dilute acetic acid (0.7%; i.p.) produced a writhing response in mice. Acetic acid treated mice showed a robust writhing response as compared with saline treated mice (81 ± 11.9 *vs* 2.2 ± 0.9, p < 0.001, n = 6, Figure 4). In contrast to writhing behavior, numbers of rearings (91.6 ± 18.8 *vs* 24.5 ± 1.5, p < 0.01, n = 6) and total distances moved (3291.6 ± 411.4 *vs* 2121.5 ± 211.9, p < 0.05, n = 6) were significantly reduced in acetic acid treated mice (Figure 4B and C). These results suggest that reduction of voluntary movements represent acute pain.

### Voluntary movements of diabetic neuropathic mice

**Figure 5:**
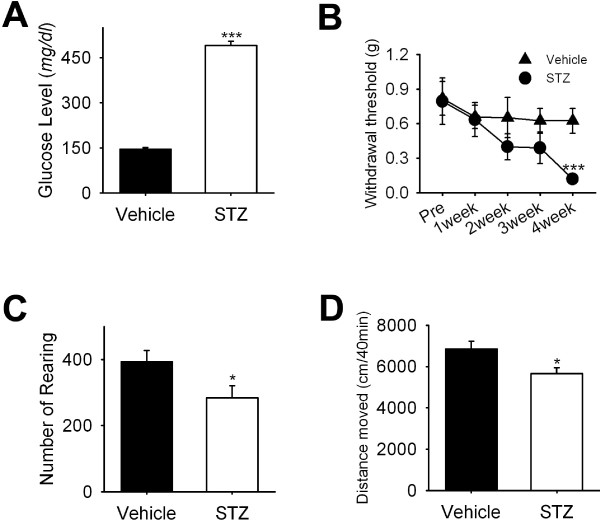
**Voluntary movements of streptozotocin-induced diabetic mice.** (**A**) Blood glucose levels of mice administered with streptozotocin (STZ) to induce diabetes. Glucose levels were measured at 4 weeks after injecting vehicle or STZ (150 mg/kg i.p.) in mice (n = 7–11). **(B)** Withdrawal thresholds to von Frey hair filaments of vehicle or STZ treated mice (n = 7–11). (**C**-**D**) Voluntary movements in vehicle or STZ treated mice. STZ treated mice showed a significant reduction in the number of rearings (**C**) and in total distances moved (**D**) (n = 7–11).

We then attempted to determine whether voluntary movements are correlated with neuropathic pain using a diabetic neuropathic pain model [19,20]. A single dose of streptozotocin (STZ) (150 mg/kg; i.p.) was used to induce diabetes. Four weeks after STZ treatment, a four-fold increase in the blood glucose level (145.7 ± 5.7 *vs* 491.3 ± 14.2 mg/dl blood, p < 0.001, n = 7–11) was observed in the STZ-treated mice (Figure 5A). To observe the time-dependent change of mechanical hypersensitivity for 4 week period, von Frey test was performed on every week in vehicle and STZ treated mice. Mechanical withdrawal threshold of STZ-treated mice was significantly reduced in 4 week after administration of STZ (Figure 5B). As was expected, diabetic mice also exhibited significant reductions in voluntary movements (Figure 5C and D).

### Voluntary movements in spared nerve injury induced mice

**Figure 6:**
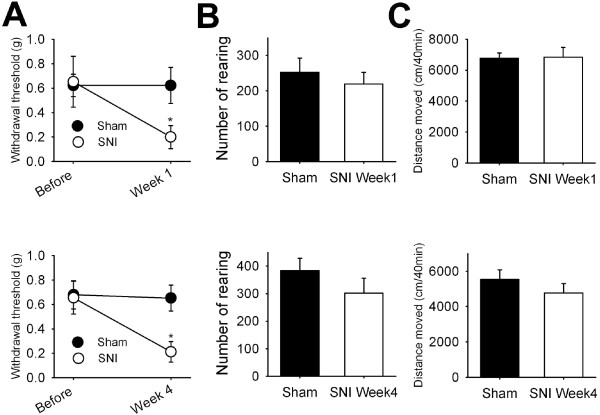
**Voluntary movements in mice with spared nerve injury (SNI)-induced neuropathic pain.** (**A**) Withdrawal thresholds to von Frey hair filaments in mice at 1 week (*top*) or 4 week (*bottom*) after surgery on sciatic nerves (SNI group) or sham operated control group (n = 9–10). **(B-C)** Numbers of rearings (**B**) and total distances moved (**C**) over 40 min were measured at 1 week or 4 week after surgery (SNI group) or sham control groups (n = 9–10).

Next, we examined whether voluntary movements could be applied to evaluate another type of neuropathic pain model, spared nerve injury (SNI) model [12]. SNI was induced by a surgical procedure that cut 2 out of 3 branches of the sciatic nerve of a rat (see Methods). Mechanical allodynia was measured in sham-operated and SNI groups at 1 week and 4 week after surgery (Figure 6). At 1 week after surgery, the SNI group resulted in a significant decrease in mechanical withdrawal threshold compared to the sham control group (0.65 ± 0.19 *vs* 0.23± 0.09g, p < 0.05, n = 9–10). The reduction in mechanical threshold was still observed in the SNI group at 4 week after surgery (0.66 ± 0.13 *vs* 0.21 ± 0.08g, p < 0.05, n = 9–10). In contrast, the number of rearing and the total distance moved of the SNI group rats were not different from those of the sham control group at 1 week or 4 week after surgery. These results indicate that the voluntary movements do not represent some types of chronic pain such as a SNI neuropathic model.

### Voluntary movements in mice lacking TRPV1

**Figure 7:**
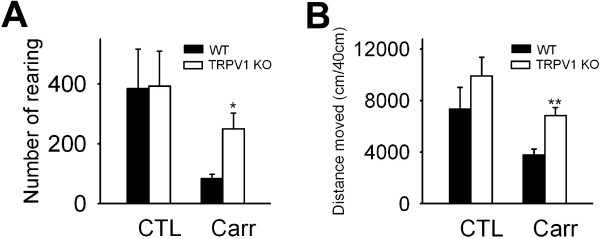
**The contribution of TRPV1 to voluntary movements in a carrageenan-induced pain model.** (**A**) Numbers of rearings over 40 min were measured at 4 h after saline (CTL) or carrageenan (Carr) injection in wild type mice and TRPV1 KO mice (n = 4–8). (**B**) Total distances moved over 40 min were measured at 4 h after saline (CTL) or carrageenan (Carr) injection in wild type mice and TRPV1 KO mice (n = 8 – 10).

Next, we then applied the voluntary movement parameters to a genetically deficient-mice model. TRPV1 is highly expressed in nociceptors and activated by capsaicin, heat and other endogenous lipids [1,21,22]. Although mice lacking TRPV1 elicit reduced inflammatory thermal hyperalgesia when tested with reflexive pain test models [23,24], it is not known whether TRPV1^-/-^ mice show reduced pain behavior when tested with non-reflexive pain tests. Therefore, we examined whether TRPV1^-/-^ mice would show change in voluntary movements as much as that of the wild-type mice. Before carrageenan was administered, wild-type and TRPV1^-/-^ mice showed similar voluntary movements, such as rearings and total distance travelled. However, when inflammation developed at 4 hrs after carrageenan injection, wild-type mice showed a marked reduction in rearings and total distance travelled, whereas the reductions in rearings and total distance travelled of TRPV1^-/-^ mice were significantly less than those of the wild-type mice (Figure 7A and B). Thus, parameters in voluntary movement can be useful to determine pain severity in genetically-modified mice.

## Discussion

In the present study, we examined the use of number of rearings and total distance moved as measures of pain, and compared the results obtained with those of reflex-based pain tests. Both parameters were found to be associated with pain intensity because they decreased in-line with paw-withdrawal latency from radiant heat in the carrageenan-induced inflammatory pain model, and in-line with withdrawal threshold as determined using von Frey filaments in a neuropathic pain model. More importantly, reductions in both parameters were reversed by NSAIDs and morphine. These results suggest that these two parameters could be used to evaluate pain intensity.

Most of pain tests are based on reflexes evoked by various noxious or non-noxious stimuli. These reflex-based pain tests have been widely used as tools for pain measurements in pre-clinical study. Although these reflex-based pain tests are useful to determine intensities of various types of pain, these pain tests may have some limitations, especially for evaluating complex and subjective pain states of patients [25]. Many approaches have been made to develop non-reflexive pain measurements. For example, Matson and colleagues suggested that reduction in the number of rearing may indicate inflammatory pain in knee joints [26]. Peripheral inflammation reduces voluntary wheel running or voluntary movements in mice or rats [27,28]. Hypolocomotion after knee surgery has been used to evaluate postoperative pain [29]. In line with these reports, the present study further supports the eligibility of voluntary movement as a useful tool for pain intensity assessment. However, in some chronic pain models, mechanical hypersensitivity does not match with hypolocomotion [30]. For example, mechanical hyperalgesia induced by SNI lasts for several weeks, whereas locomotion is not different between injured and naïve animals [30]. In accordance with this, in this study, number of rearing and total distance moved in SNI group were not different from those of shame control group. Interestingly, voluntary movements of mice with chronic constriction injury on sciatic nerve are reduced [30]. Moreover, diabetic neuropathic pain model in this study showed a significant reduction in voluntary movements as well as mechanical threshold (Figure 5). Thus, voluntary movements may represent some types of pain, not all types of chronic pain.

Another concern regarding use of non-reflexive pain tests is the location of injury. If the injury is given to lower limbs, it seems difficult to judge whether the reduction in voluntary movement is caused by the difficulty in physical motion or the reluctance of motion due to perception of pain. To answer this question, we applied noxious stimulus, acetic acid, to peritoneum. The acetic acid-treated mice exhibited reduced voluntary movements (Figure 3). Thus, change in voluntary movement may represent the intensity of various types of pain.

The merits of using voluntary movements to assess pain are many folds. First, because animals are placed in a relatively large chamber they are able to move freely without physical restriction, which avoids the stress caused by restricting holding animals, which could affect pain sensitivity [26,31]. Second, the testing is easily performed and is less subjective than conventional tests because measurements are recorded automatically. Third, voluntary movements involve cerebral components of pain perception and possibly exclude components of spinal nociceptive reflexes. Fourth, the testing does not need habituation because it utilizes the exploratory behavior. Finally, the testing covers various types of pain models, such as acute as well as inflammatory and neuropathic pain.

The present study also shows that observed reductions in voluntary movements were significantly reversed by common analgesic drugs, such as ibuprofen, diclofenac, celecoxib and by morphine, which indicates that measurements of voluntary movements provide a means of evaluating the analgesic efficacies of non-steroidal anti-inflammatory drugs or opiates. Some analgesics cause sedation [32]. For example, morphine is well known to act as a sedative at higher doses [26,33,34], and therefore, it is important to distinguish between analgesic and adverse cognitive effects when evaluating analgesic activities. However, classical pain tests often produce false-positive results for drugs with a sedating effect, because analgesic responses and sedative responses cause conventional pain tests to move in the same direction. But this is not the case for voluntary movement testing: an increase in voluntary movement indicates an analgesic effect whereas a decrease indicates a sedative effect. Thus, the use of voluntary movement testing is likely to reduce false-positive results for analgesics [26,34].

Summarizing, voluntary movement measurements were found to provide measured of some types of chronic pain. We believe that application of this type of testing during basic studies or screening candidate drugs would contribute to our understanding of pain mechanisms and thus to the development of new analgesics.

## Conclusions

In summary, our results show that voluntary movements are effective and easy measurements for evaluating intensity for various types of pain. Therefore, they can be useful tools for screening candidate chemicals for novel analgesics.

## Abbreviations

(Carr): Carrageenan; (STZ): Streptozotocin; (Ibupro): Ibuptofen; (Morph): Morphine; (CTL): Control; (WT): Wild-type; (KO): Knock-out.

## Competing interests

The authors declare that they have no competing interests.

## Author’s contributions

HC and YJ designed the study, and performed behavioral experiments. HC, SMK and SWH performed behavioral experiments. BL measured glucose level in animals. UO coordinated the study and prepared the paper. All authors read and approved the final manuscript.
